# Influence of pre-admission factors on quality of life and adaptation in nursing home residents with dementia: the QOL-EHPAD study protocol

**DOI:** 10.1186/s12877-020-1434-2

**Published:** 2020-03-06

**Authors:** Roxane Villeneuve, Céline Meillon, Valérie Bergua, Maturin Tabue-Teguo, Hélène Amieva

**Affiliations:** 1 Inserm U1219 Bordeaux Population Health Center, Université de Bordeaux, 33076 Bordeaux, France; 2 Service de Gériatrie, CHU de Pointe-à-Pitre, Université des Antilles, Pointe-a-Pitre, Guadeloupe France

**Keywords:** Dementia, Predictors, Quality of life, Adaptation, Nursing home

## Abstract

**Background:**

In 2015 in France, 585,560 people were nursing home residents. A large body of studies has identified predictors of poor quality of life and poor adaptation in institution, mostly for residents without dementia. With 42 to 72% of these residents diagnosed with dementia, it is crucial to identify what factors prior to admission might have an impact on quality of life once the admission is finalized, in order to target specific domains of intervention, while the person still lives at home and after his/her admission.

**Methods:**

QOL-EHPAD is a prospective, multi-centred, observational cohort study. At baseline, we will collect retrospective data on the life of 150 persons with dementia and their caregivers. These data will refer to the conditions of admission to a nursing home (emergency admission, involvement in the decision, admission from home or from the hospital) and to the 6 months prior to the admission of the person with dementia: sociodemographic and medical data, psychological tests, information on quality of life, satisfaction, behaviour, and nutrition. Similar data about life in the nursing home will be collected after 6 months, along with information on adaptation of the person with dementia to his/her new living environment. We will use univariate regression analyses followed by stepwise linear regression models to identify which factors pertaining to life at home are associated with quality of life and adaptation after 6 months.

**Discussion:**

This study will provide data on the impact of institutionalization on quality of life and the determinants of a successful institutionalization in people with dementia. This could be helpful in setting up targeted interventions to prepare admission into a nursing home before the actual admission and to accompany both the caregiver and the person with dementia throughout this process.

## Background

In 2015, 585,560 older people were living in a nursing home in France [1]. Beyond age, several risk factors are known to predict nursing home admission: being a woman, living alone, lower income, falls, a high number of visits to a medical specialist, poor family relationships and dependency [2–6]. This latter has become the most common feature of nursing home residents, half of them presenting both Activities of Daily Living (ADL) and Instrumental Activities of Daily Living (IADL) disability [7].

Nursing home admission requires the resident to adapt to a new living environment, thus to new rhythms and routines, shaped by the constraints of group living. A good adaptation is positively influenced by having good physical autonomy and feeling prepared to enter the institution [8]. However, in 2000, only 35% of the residents declared they actively participated in the admission process, the main actors being their relatives [9]. This feeling of being dispossessed of their choices might greatly affect quality of life [10]. Quality of life is a multidimensional construct, with both objective and subjective indices. According to the World Health Organisation, it is the “perception of individuals of their position in life in the context of the culture and value systems in which they live, and in relation to their goals, expectations, standards and concerns”. The question of adjustment and quality of life in nursing homes is particularly relevant for people with dementia, who represent more than half of nursing home residents [11]. Adaptation to any change may require substantial cognitive resources [12] and quality of life relies on one’s appreciation of what one has. In addition, assessing quality of life might prove challenging in persons with cognitive impairment.

It is unclear whether entering a nursing home affects the quality of life of people with dementia, the literature providing inconsistent results. Indeed, while some studies suggest that quality of life is not impacted by admission into a nursing home [13, 14], others show a significant decrease in quality of life scores [15]. Other studies are less clear-cut: in Missotten et al. [16], no difference in quality of life was observed between baseline and 2 years later. Nonetheless, there were fluctuations in quality of life scores over this period suggesting high variability between individuals and over time. The abundance of quality of life scales for people with dementia may partly explain such discrepancies in results, especially since many of them are based on different theoretical backgrounds (see Missotten et al., [17] for a review of quality of life questionnaires in dementia). Comparison between study results is thus challenging.

In people with dementia living in nursing homes, a higher number of chronic diseases, the presence of sensory impairments and neuropsychiatric symptoms, higher quality of life at the time of admission, as well as the quality of life of the caregiver, are associated with lower quality of life and a negative evolution of quality of life over time [13, 14, 18–20]. The impact of altered cognitive functioning on quality of life in dementia remains debated, and seems to negatively impact proxy-ratings of quality of life but not self-rated evaluations [21]. On the other hand, participation in leisure activities and social satisfaction are consistently associated with higher quality of life [22, 23]. Nevertheless, all these studies are interested in quality of life from the time of admission and do not consider previous factors about the situation at home before admission, yet these factors might prove significant. For example, Achterberg [24] shows that 10 days after being admitted into a nursing home, demented people exhibit less depressive symptoms when they are admitted from a hospital than when they are admitted directly from their home, underlying the importance of previous housing characteristics.

We have thus identified four types of pre-admission factors that might affect both quality of life and adaptation of nursing homes residents diagnosed with dementia:

- sociodemographic and clinical characteristics of both the resident and his/her caregiver such as sex, age, level of education, mean income, cognition, dependency (difficulties in ADL and/or IADL), comorbidities, depression [8, 9, 25];
- social environment before admission: living conditions such as living alone, participation in social activities, perceived social support [26];
- involvement in the decision to enter a nursing home [8];
- conditions of admission: emergency or programmed admission, hospital stay before admission [9, 24, 27].

### Objective

The objective of the QOL-EHPAD (Quality of Life in French Nursing Homes – Etablissement d’Hébergement pour Personnes Agées Dépendantes) study is to assess the impact of clinical characteristics, living conditions and environment before admission, as well as conditions of admission (i.e. emergency / programmed admission and decision-making conditions regarding nursing home admission) on quality of life and adaptation of people with dementia after 6 months in institution.

#### Primary hypothesis

- Emergency admission and/or admission without the person with dementia being consulted is associated with a lower score of quality of life measured with the Quality of Life - Alzheimer’s Disease scale (QOL-AD, [28, 29]) six months after admission.

#### Secondary hypotheses

- Emergency admission and/or admission without the person with dementia being consulted is associated with a lower adaptation score six months after admission.
- Higher score of quality of life in the six months before admission is associated with lower quality of life and adaptation scores after six months after admission.
- Higher number of comorbidities during the six months before admission is associated with lower quality of life and adaptation scores six months after admission.
- Higher score of behavioural disturbance in the six months before admission is associated with lower quality of life and adaptation scores six months after admission.
- Higher perceived social support in the six months before admission is associated with higher quality of life and adaptation scores six months after admission.
- Higher participation in social activities in the six months before admission is associated with lower quality of life and adaptation scores six months after admission.
- Higher level of routines in the six months before admission is associated with lower quality of life and adaptation scores six months after admission.
- Depressive symptoms in the six months before admission are associated with lower quality of life and adaptation six months after admission.
- Sensory impairment before admission is associated with lower quality of life and adaptation six months after admission.
- Higher cognitive functioning in the six months before admission is associated with lower quality of life and adaptation scores six months after admission.
- Lower quality of life of caregivers in the six months before admission is associated with lower quality of life and adaptation scores of residents with dementia six months after admission.

## Methods/design

### Recruitment process and inclusion criteria

QOL-EHPAD is a prospective, multi-centred, observational cohort study. We intend to recruit 150 dyads (participant with dementia and his/her caregiver) from 14 public nursing homes located in a French administrative area of Southwestern France (Lot-et-Garonne). This area combines both rural and urban settings, which will allow for a wide exploration of home situations. Additional nursing homes may be included if necessary in order to reach the target sample size of 150 dyads, and bimonthly contact is established with a referent from each nursing home to review admissions and possible inclusions.

Before the inclusion visit, the nursing home physician goes over the admission files of the future residents or newly admitted residents to review eligibility criteria. If the profile fits, s/he sets up a face-to-face or a phone interview to ask for consent to both the person with dementia and his/her caregiver. S/he then reviews the inclusion and exclusion criteria with them. S/he gives them information on the nature and objective of the research and on the risks and benefits associated, as well as on the confidentiality agreement. S/he provides both caregiver and person with dementia with an information notice and keeps a copy for the investigator file. Due to the observational nature of the study, the Ethics Committee does not require consent forms. Nevertheless, the physician signs and dates each information notice for traceability of the agreement to participate. The physician then contacts the psychologist who will conduct interviews. The psychologist then sets up interviews with the participants. All questionnaires will be administered by a trained psychologist. Delay between admission and time of the interview should not exceed a month, considering the time-sensitive nature of the collected information.

Table 1 shows the inclusion and exclusion criteria for the resident and the caregiver. The main criteria for the resident are to be diagnosed with dementia according to DSM-IV criteria and being admitted to one of the nursing homes collaborating to the project. The diagnostic process falls outside the scope of our study, and diagnosis of dementia must be established by a specialist beforehand. The main inclusion criterion for the caregiver is to have sufficient knowledge of the person with dementia to provide accurate information about the situation of the person before admission. The caregiver may be a relative or a friend of the resident. In the absence of an identified informal caregiver, s/he may also be a professional caregiver who has known the participant for more than a year.

**Table 1:** Inclusion and exclusion criteria of the QoL-EHPAD study

	Person with dementia	Caregiver
Inclusion criteria	- 65 years old and older - Diagnosis of dementia according to DSM-IV criteria - Programmed admission to one of the nursing homes - collaborating to the project - Caregiver willing to participate - Agrees to participate	- 18 years old and older - Interacts with the participant with dementia at least once a week. - Has known the participant for more than a year - Is not opposed to participating
Exclusion criteria	- Unstable, acute or current psychiatric or physical condition severe enough to prevent them from participating in the study - Under tutorship - Benefits from end-of-life care	- Unstable, acute or current psychiatric or physical condition severe enough to prevent them from participating in the study - Under tutorship

### Measurements

Baseline data are retrospective and refer to the six-month period before admission. Caregivers and persons with dementia are interviewed separately. The six-month follow-up requires data collection from informal caregivers, nursing home caregivers and persons with dementia. Table 2 shows a summary of the information collected throughout the study. A validated French version was available for every scale we used in the study.

**Table 2:** Summary of data collected in the QoL-EHPAD study

	Pre-admission information collected at baseline	Six-month post-admission visit
Primary outcome	Quality of life: QOL-AD	Quality of life**:** QOL-AD
Secondary outcomes		Adaptation**:** Adaptation to the institution scale
Determinants		
Sociodemographic and medical data		
	Age, sex, level of education Age, sex, level of education - caregiver Personality: TIPI Medication Medical history and comorbidities Functional status: ADL / IADL Cognition: MMSE Behavioural disturbances: NPI Nutrition: MNA Depression: CES-D Depression – caregiver: CES-D Burden – caregiver: Zarit	Medication Comorbidities Functional status: AGGIR Cognition: MMSE Behavioural disturbances: NPI Nutrition: MNA Depression: CES-D Depression – caregiver: CES-D Burden – caregiver: Zarit Satisfaction
Social environment		
	Satisfaction Satisfaction - caregiver Participation in activities Quality of life - caregiver: SF-12 Routines: échelle de préférence de routinisation	Satisfaction - caregiver Participation in activities Quality of life - caregiver: SF-12
Decision process		
	Reason for admission Decision makers Involvement in the decision Agreement with the decision	
Conditions of admission		
	Emergency/planned admission Place of stay before admission	

*QoL-AD* Quality of life – Alzheimer’s Disease, Activities of Daily Living /Instrumental Activities of Daily Living, *SF*-12 Short form survey – 12 items; *CES-D* Center for epidemiologic studies – depression, *MMSE* Mini mental state examination, *MNA* Mini nutritional assessment, *NPI* Neuropsychiatric interview, *TIPI* Ten item personality inventory

#### Primary outcome

##### Quality of life

Resident’s quality of life is assessed using the Quality of Life in Alzheimer’s Disease (QOL-AD, [28, 29]) one of the most widely used quality of life scale in dementia research [30]. The QOL-AD offers a brief yet reliable evaluation of quality of life, even in severe dementia [31], thanks to its sound psychometric properties. It shows good internal consistency, responsiveness and content validity [32, 33]. It explores several aspects of quality of life, such as social relationships, health, well-being, leisure, memory, and mood. Scores for each item range from « poor » (1) to « excellent » (4), while total score ranges from 13 to 52. At baseline, the questionnaire is filled out by the caregiver and the person with dementia; at follow-up, by the person with dementia, their caregiver and the designated professional caregiver.

#### Secondary outcome

##### Adaptation

Adaptation to the nursing home is assessed after six months using the French Scale of Adaptation to the institution [34]. This 17-item questionnaire includes statements about daily life, habits, and social relationships. The person with dementia has to say whether the statement applies to his/her current situation. A score equal to or over 16 tends to indicate successful adjustment, while it indicates great difficulties with adapting if equal to or under 11.

#### Determinants of quality of life and adaptation

##### Socio demographic and medical data

A number of sociodemographic data about the person with dementia and his/her caregiver are collected: sex, age, level of education, housing situation, marital status, financial support as well as medical data of the person with dementia (medical history, drugs consumption).

##### Meaningful activities

Data on daily and leisure activities are provided by the caregiver. S/he has to say whether the person with dementia performed activities at home that bore meaning to him/her. Then, the type and number of meaningful activities are collected. This type of data collection allows any type of activity to be included, whether solitary or social.

##### Life satisfaction

Participants evaluate their satisfaction with their life as a whole, with their health and with their friends and family using single items.

##### Quality of life of the caregiver

To assess quality of life of the caregiver at baseline and follow-up, we use the short version of the Medical Outcomes Study Short-Form General Health Survey (SF-36 [35]), the SF-12 [36]. Widely used in epidemiology, it assesses quality of life in link with physical and psychological health.

##### Behavioural disturbance

Presence, frequency, severity and impact on caregivers of behavioural and cognitive symptoms of the person with dementia are assessed with the Neuropsychiatric Inventory (NPI [37]) at baseline and after six months.

##### Caregiver’s burden

Caregiver’s burden is assessed at baseline and after six months using the Zarit Burden Interview [38]. With a total score ranging from 0 (no burden) to 88 (severe burden), this 22-item questionnaire explores the impact of several dimensions of care (social, emotional, financial, etc.) on the caregiver.

##### Nutritional state

Nutritional state of the person with dementia is assessed at baseline and after six months using the Mini Nutritional Assessment [39]. This two-part questionnaire allows detecting malnutrition. If the score is inferior to 11, the examiner proceeds with the second part for an in-depth evaluation.

##### Dependency

Three questionnaires assess dependency. At baseline, caregivers evaluate the person with dementia’s autonomy at home. We use the Katz Index of Independency in ADL [40], a six-items scale assessing abilities in basic acts of everyday life (such as bathing, eating or dressing up). The Lawton IADL scale [41] allows for the evaluation of abilities in instrumental activities(e.g. using the phone, shopping, etc).

At follow-up, the designated professional caregiver evaluates dependency using the AGGIR questionnaire (*Autonomie Gérontologie Groupes Iso Ressources*). This 17-item French-specific grid classifies people into six groups according to their degree of dependency in ten bodily and mental functions and seven activities (these “illustrative” activities are not included in the final score). A score of 6 indicates total autonomy while 1 indicates total dependency.

##### Personality

With the Ten Item Personality Inventory (TIPI) [42], caregivers are asked at baseline to evaluate the personality traits of the person with dementia, in accordance with the Big Five theory (Openness, Conscientiousness, Extraversion, Agreeableness and Neuroticism).

##### Routinization

Filled out by the caregiver at baseline, the short version of The French Routinization Scale [43] evaluates routinization preferences of the person with dementia through six questions about everyday habits. The higher the score (out of 30), the more the person is routinized.

##### Cognition

Global cognitive functioning is assessed with the Mini-Mental State Examination [44], a 30-item summed score used as an index for global cognitive performances. The total score ranges from 0 to 30.

##### Depression

The Center for Epidemiologic Study - Depression Scale (CES-D [45, 46]), a 20-item questionnaire, is used to assess depressive symptomatology at baseline and after six months. With a score ranging from 0 to 60, persons with dementia have to assess the frequency of their symptoms, while caregivers assess their own symptoms and the person with dementia’s.

##### Decision making

Involvement of the person with dementia and his/her caregivers and relatives in the decision to enter a nursing home are collected from the caregiver. We will also explore whether the person with dementia agreed with this decision with a dichotomous question.

##### Admission

Data on conditions of admission are collected: was it an emergency or planned admission? Place of stay before admission is also collected. We ask whether the participant with dementia was admitted from his own home, from a hospital or from any temporary housing.

### Sample size

In order to calculate the target sample size, we used data from Castro-Monteiro et al. [19]. They found a mean difference in QOL-AD scores between admission and after 6 months of 1.7 point in people with dementia. Therefore, we considered this difference in QOL-AD scores as clinically significant. To observe such a difference in our study, we need a sample size of 130 subjects, with a 90% statistical power and a 5% maximum probability of type 1 error alpha. This sample size was calculated using the Rstudio software (power.t.test), and was rounded to 150 to account for attrition, some studies showing an attrition rate of about 30% after 3 months [47].

### Statistical analyses

#### Inclusion and follow-up data

First a description of number of participants, inclusion rhythm (evolution of inclusion from the first to the last inclusion), expected number of visits and actual number of visits, violations to the protocol if any, and causes for attrition, will be performed.

#### Participants’ characteristics at baseline and follow-up

At baseline and follow-up, categorical data will be described as follows: frequency, number and percentage. We will compare baseline data of people who answered after 6 months with data of people lost to follow-up using a chi-square test.

At baseline and follow-up, continuous data will be described as follows: mean, standard deviation, minimum, maximum and interquartile intervals. We will compare baseline data of people who answered after 6 months with data of people lost to follow-up using a Student T test.

#### Primary and secondary outcomes

Statistical analyses will be similar for both primary and secondary outcomes.

Scores will be described at baseline and after 6 months for the QOL-AD, and after 6 months for the Adaptation to the Residence scale as follows: mean, standard deviation, minimum, maximum and interquartile intervals.

We will perform linear regression models to assess the association between the variables and our outcomes. If *p < 0.20,* variable will be included in a stepwise regression model, after we check for multicollinearity with a correlation matrix. We will then use a backward stepwise regression model to identify the best predictors of quality of life and adaptation (*p <* 0.05). All statistical analyses will be performed using Rstudio [48].

### Data management

Data management and statistical analyses (data entry, cleaning, handling of missing data) will be performed by the SEPIA Team, from the Bordeaux Population Health Research Centre. Data will be anonymized, password-protected and stored on a secure server. No transfer of data will be authorized.

### Auditing and monitoring

The sponsor (Agen-Nérac Hospital Centre) is allowed to carry out an audit at any stage of the research. This audit is decided and performed independently from the investigators. The audit consists in checking participants’ safety, respect of participants’ rights, compliance with research regulations, and data reliability. A steering committee has been set up. Its role is to provide overall supervision of the research and monitor the progress of the trial in accordance with the protocol. A more restricted trial management group including the principal investigator ensures the day-to-day study management. The data monitoring management is carried out by an independent data manager. The study is conducted in accordance with the declaration of Helsinki and with the 2012–300 law regarding research involving humans.

### Publication and dissemination policies

Every 2 months, the field partners receive a newsletter giving updates on inclusions and amendments to the protocol, if any. Results will be published in international scientific journals and presented in meetings (in partner nursing homes) and in scientific congresses.

Authorship status for communications on this study will comply with standard authorship guidelines, which guarantee substantial contribution. No professional writer will participate.

## Discussion

Comprehensive research on the quality of life of people with dementia throughout the transition between home care and nursing home remains scarce. We can reasonably think that our study will contribute to identify which factors, before admission, predict quality of life and adjustment to nursing home. This will contribute to a better understanding of the impact of institutionalization on quality of life and the determinants of a “successful” nursing home admission.

Given the time and memory sensitive nature of the data, main analyses on living conditions at home will be performed using caregivers’ data. Comparison between the caregivers’ and person with dementia’s data in further analyses will offer interesting insight on how a person with dementia reconstructs the idea of life at home, and if this reconstruction reflects the objective conditions of her daily life and the perception of the main caregiver. The evaluation by three different persons of the resident’s quality of life with the QoL-AD – by the resident him/herself, his/her relative and nursing home carer - will also allow for cross-sectional analyses and comparisons of predictors according to the evaluator.

To conclude, we need robust data on predictors of quality of life and adaptation in people with dementia before implementing any kind of intervention dedicated to promote well-being. The existing interventions mostly start at admission [49] or focus on delaying admission while still at home [50]. By identifying the critical factors influencing adaptation and quality of life after entering a nursing home, this study should provide information on how to optimize the transition between pre and post-placement, especially when admission becomes the only viable option for the person with dementia and his/her caregiver. This research should be helpful for the implementation of targeted interventions to prepare admission into a nursing home, for both the caregiver and the person with dementia, according to their profile before the admission.

## Data Availability

The datasets used and/or analysed during the current study will be available from the corresponding author on reasonable request. (RV).

## Abbreviations

AGGIR: Grille iso-ressources
CES-D: Center for epidemiologic studies – depression
MMSE: Mini mental state examination
MNA: Mini nutritional assessment
NPI: Neuropsychiatric interview
QoL-AD: Quality of life – alzheimer’s disease
SF-12: Short form survey – 12 items
TIPI: Ten item personality inventory
